# Dynamics and Health Risks of Fungal Bioaerosols in Confined Broiler Houses During Winter

**DOI:** 10.3390/ani16030437

**Published:** 2026-01-30

**Authors:** Mengxi Yan, Zhuhua Liu, Mingli Liu, Huage Liu, Zhenyue Li, Zitong Yang, Yi Lu, Wenhao Feng, Xiaolong Chen, Shuang Cheng, Yuqing Yang, Cheng Zhang, Xuejing Wang, Huan Cui

**Affiliations:** 1 College of Veterinary Medicine, Hebei Agricultural University, Baoding 071000, China; 2 The Animal Husbandry and Veterinary Institute of Hebei, Baoding 071001, China; 3 College of Animal Science and Technology, Hebei North University, Zhangjiakou 075000, China

**Keywords:** fungal bioaerosols, particle size distribution, airborne microbiome, One Health, winter confined environment

## Abstract

Fungal bioaerosols play a critical ecological and health role in intensive poultry production systems. However, their dynamic characteristics and community succession patterns in confined cage environments during winter remain poorly understood. This study investigated a typical confined broiler house in Hebei Province, China, during winter. A combined approach of Andersen six-stage sampling, colony counting, and Internal Transcribed Spacer (ITS) high-throughput sequencing was employed to comprehensively analyze the concentration, particle size distribution, diversity, and community composition of fungal bioaerosols across three key growth stages: 7 days (brooding phase), 21 days (growing phase), and 35 days (finishing phase). The results revealed a significant increasing trend in fungal aerosol concentration as the rearing cycle progressed, increasing from 1125 ± 125 CFU/m^3^ at day 7 to 3872 ± 565 CFU/m^3^ at day 35 (*p* < 0.001), reaching high-risk exposure levels in the later stages. Small-sized fungal bioaerosols (<4.7 μm) were dominant across all stages (54.35–65.50%), with the highest proportion observed at day 21, indicating their potential for deep respiratory deposition and long-distance airborne transmission. The number of Operational Taxonomic Units (OTUs), along with Chao1 and Shannon indices, increased significantly with bird age (*p* < 0.001), demonstrating a clear community succession from early-stage yeast-dominated forms (e.g., *Diutina*, *Blumeria*) to mid- and late-stage assemblages dominated by filamentous fungi (e.g., *Aspergillus*, *Cladosporium*, *Alternaria*). Notably, several zoonotic pathogenic genera were detected throughout all rearing stages, highlighting the potential risks of airborne fungi to animal health, occupational exposure, and environmental safety under winter ventilation restrictions. This study characterizes a stage-dependent pattern of increasing airborne fungal concentrations accompanied by shifts in particle size distribution and community composition under winter confined conditions. The findings provide a crucial scientific basis for optimizing winter ventilation and environmental management strategies, improving environmental control technologies, establishing airborne biosafety standards, and developing targeted fungal monitoring and prevention technologies.

## Simple Summary

Airborne fungi are an important but often overlooked component of the indoor environment in modern poultry production, especially during winter when ventilation is intentionally reduced to maintain warmth. This study monitored fungal particles in enclosed broiler houses at three key growth stages to understand how their levels, size characteristics, and species composition change over time. We found that fungal concentrations increased markedly as the birds aged, and that small particles capable of reaching deep into the lungs dominated throughout the production cycle. The types of fungi also shifted from early-stage yeasts to later-stage filamentous species, including several known to cause respiratory irritation or disease. These findings highlight that winter housing conditions can create a progressively higher fungal exposure risk for both poultry and workers. The results provide scientific evidence supporting improved ventilation management, hygiene practices, and environmental monitoring to reduce airborne fungal risks in intensive poultry systems.

## 1. Introduction

Intensive poultry production systems are vital to modern animal agriculture, playing a central role in ensuring a stable supply of animal protein and promoting sustainable agricultural development. However, while enhancing production efficiency, the confined and high-density rearing environments also exacerbate airborne microbial pollution. Among these pollutants, fungal bioaerosols, as a significant component of airborne pathogens, have emerged as a critical environmental risk factor threatening flock health, occupational safety for farm workers, and public health [1,2,3]. Characterized by strong airborne stability and wide dissemination range, fungal bioaerosols can enter the host’s respiratory tract and even deep alveoli via inhalation, triggering respiratory infections, immunosuppression, and growth retardation in poultry. Simultaneously, they pose a continuous threat to farm workers by potentially causing zoonotic fungal diseases [4,5].

In poultry production systems, fungal proliferation and dispersal are closely associated with feed spoilage, and the accumulation of bird secretions and manure [1]. Their spores become aerosolized through air turbulence from ventilation and bird activity. This is particularly pronounced in winter confined-cage systems, where ventilation is often restricted to maintain suitable indoor temperature and humidity. This management strategy results in reduced air velocity, dust accumulation, and fluctuating temperature/humidity, significantly increasing the release and retention of fungal spores [6,7,8]. Size-fractionated sampling has revealed that the airborne suspension time of fungal spores can be prolonged by 3–5 times under low-temperature and low-humidity conditions [9]. Previous studies indicate that fungi in poultry house air form complex “bio-particle complexes” with particulate matter (PM_2.5_/PM_10_), further enhancing the aerosol’s dispersion stability and environmental adaptability [7].

The size characteristics of aerosolized fungi are decisive for their deposition velocity and health risks. Existing research has established that spores smaller than 4.7 µm are respirable and can penetrate deep into the host’s alveoli, inducing various diseases such as chronic respiratory inflammation, asthma, and aspergillosis [10,11,12]. Studies on Portuguese poultry farms confirmed that small-sized fungi (in the PM_2.5_ range) can constitute over 70% of the total spore count in ventilation-restricted environments [1]. Furthermore, aerodynamic properties influence fungal transmission distance and cross-host infectivity [13]. Field measurements have demonstrated that airflow patterns and air humidity jointly determine the size distribution and vertical migration pathways of fungi within poultry houses, highlighting the unique risk profile of fungal aerosols in winter confined environments [6].

Globally, research on airborne microorganisms in poultry houses has predominantly focused on bacterial aerosols, whereas systematic studies on fungal communities remain relatively limited [14]. Investigations in Europe and North America have shown that fungal concentrations in poultry house air typically range from 10^2^ to 10^4^ CFU/m^3^, with *Aspergillus*, *Penicillium*, *Cladosporium*, *Alternaria*, and *Candida* being the most frequently detected genera [1,5,15]. Studies in intensive chicken farms in Northern China have identified abundant fungal spores and antibiotic resistance genes (ARGs) in airborne PM_2.5_, suggesting that fungi may serve as vectors for ARG dissemination [4]. Concurrently, high-throughput sequencing has verified that the structure of airborne fungal communities in poultry houses is co-regulated by multiple factors, including humidity, stocking density, and feed formulation [3,16,17]. Moreover, airborne fungal pollution is closely linked to poultry production performance and immune function; high concentrations of fungal spores can induce immunosuppression and growth retardation in chickens, while long-term exposure may lead to decreased egg production in layers and increased feed conversion ratios [12]. Previous research found significantly higher airborne fungal diversity in caged hen houses in Northern China during the cold season compared to the warm season, with low ventilation during the cold season hypothesized as the key environmental driver [5]. Collectively, these studies indicate that airborne fungal contamination in poultry houses has become a significant factor affecting animal health, production efficiency, and environmental safety.

The application of Internal Transcribed Spacer (ITS) high-throughput sequencing technology enables comprehensive profiling of the structure, abundance, and succession patterns of airborne fungal communities [3,18,19]. Notably, the ecological diversity of airborne fungal communities not only determines their environmental adaptability but is also directly linked to pathogenic risk. Research has found that airborne fungal communities in poultry houses are typically composed of genera such as *Aspergillus*, *Candida*, *Fusarium*, *Alternaria*, *Cladosporium*, and *Trichoderma*. These fungi can not only cause respiratory infections in poultry but also lead to occupational diseases like fungal pneumonia and allergic alveolitis in farm workers through occupational exposure [13,19,20]. Multiple drug-resistant fungal strains, with resistance profiles similar to clinical isolates, have been detected in air samples from Egyptian broiler slaughterhouses [10]. From a “One Health” perspective, Qian et al. pointed out that fungal aerosols act as microbial transmission vectors between animals, humans, and the environment, representing a critical link in the spread of antimicrobial resistance [11]. Studies have shown that long-term exposure to air containing *Aspergillus* or *Cladosporium* spores increases the risk of chronic bronchitis, asthma, and allergic rhinitis among farm workers [19,21].

Despite a growing number of reports on fungal aerosols in poultry houses in recent years, research focusing on winter confined-cage systems in Northern China remains relatively scarce [5,6,7]. Hebei Province is a major broiler production region in China. During winter, this area experiences cold and dry climate conditions, and cage systems often require reduced ventilation rates to maintain thermal balance, creating a unique microenvironment characterized by low air velocity, high dust load, and significant humidity fluctuations. This environment may substantially alter the concentration levels, size distribution, and community composition of airborne fungi [3,7], yet related studies are currently lacking. Furthermore, existing research often lacks systematic investigation into the size distribution and community succession of airborne fungi across different broiler growth stages within the same study. There is also a scarcity of integrated analytical frameworks combining ITS sequencing technology with aerodynamic characteristics, hindering a comprehensive understanding of the ecological patterns and risk mechanisms of fungal aerosols in winter confined environments.

Therefore, this study investigates typical confined-cage broiler houses in Hebei Province during winter. It systematically analyzes, for the first time under these conditions, the concentration dynamics, size distribution characteristics, and community structural succession of airborne fungal aerosols during three key growth stages: 7 days (brooding), 21 days (growing), and 35 days (finishing). By integrating Andersen six-stage impaction sampling with ITS high-throughput sequencing, this research aims to: (1) reveal the stage-specific variation patterns of fungal aerosols in winter confined-cage poultry houses; (2) elucidate the association between aerodynamic particle size and fungal community composition; and (3) identify potential zoonotic pathogenic fungal genera. The findings will provide a scientific basis for improving airborne hygiene management, optimizing winter ventilation strategies, and preventing One Health risks associated with fungal bioaerosols. The results of this study will offer empirical support for a deeper understanding of the ecological mechanisms of fungal aerosols in livestock production environments and lay the groundwork for establishing a precise airborne biosafety assessment system and constructing an integrated health protection framework for “flock-personnel-environment”.

## 2. Materials and Methods

### 2.1. Sampling Sites and Poultry House Environment

This study was conducted during the winter of 2024 in Hebei Province, China. Based on the geographical distribution and scale gradient of broiler farming, five representative confined-cage broiler farms were uniformly selected as sampling sites. In accordance with corporate confidentiality agreements, the specific geographical locations are anonymized. All poultry houses were H-type, south–north oriented, three-tiered cage confinement structures, adapted to the local monsoon climate. The roofs and sidewalls were insulated with thermal materials to enhance temperature stability in winter. All houses were managed by the same enterprise under standardized protocols, with uniform feed formulation, vaccination programs, and daily cleaning frequency to ensure consistent rearing conditions.

The broilers were Arbor Acres (AA) strain with a 42-day production cycle, reared at a density of 20–21 birds/m^2^, with each house accommodating 28,000–30,000 birds. Air sampling was conducted strictly at 7, 21, and 35 days of age, with environmental conditions tightly controlled. Each house was equipped with an integrated environmental control system, including inlet ventilation windows, artificial lighting, heating equipment, evaporative cooling pads, automatic manure removal belts, and nipple drinker lines.

House temperature and relative humidity were dynamically regulated according to the growth phase: maintained at 31–33 °C and 65–70% during the brooding phase; 29–31 °C and 55–65% during the growing phase; and 21–22 °C and 50–60% during the finishing phase. A tunnel ventilation design was employed, where fresh air entered from front inlets and was exhausted by fans at the opposite end, creating a consistent longitudinal airflow. Under Hebei winter conditions, the ventilation rate was controlled at 0.2 m/s to maintain thermal balance. Manure was regularly removed using an automated scraping system to mitigate its impact on the airborne microbial community. Furthermore, all houses underwent additional thorough cleaning and disinfection 24 h prior to air sampling to ensure that the collected samples reflected differences attributable to flock growth stage and environmental conditions.

### 2.2. Determination of Fungal Aerosol Size Distribution and Concentration

The size distribution and concentration of colony fungal aerosols were measured using an Andersen six-stage viable microbial sampler (Model ZR-2001, Qingdao Zhongrui Instrument Co., Ltd., Qingdao, China), following the manufacturer’s standard operating instructions. The impactor separates particles into six stages based on aerodynamic diameter, as follows: >7.0 µm, 4.7–7.0 µm, 3.3–4.7 µm, 2.1–3.3 µm, 1.1–2.1 µm, and 0.65–1.1 µm. Sampling points were located at the front, middle, and rear positions along the longitudinal central axis of the house, approximately 26 m apart, with the sampler inlet positioned at a height of 1 m above the floor [15]. At each point, sampling was conducted at a flow rate of 28.3 L/min for 2 min, with five parallel samples collected per location.

Rose Bengal Agar (HB8643, Shanghai Haibo Biotechnology Co., Ltd., Shanghai, China) supplemented with chloramphenicol (C0378, Solarbio, Beijing, China) to inhibit bacterial growth was used as the collection medium [22]. After sampling, plates were incubated immediately at 28 °C for 5–7 days before fungal colonies were counted. Results were corrected using the positive hole conversion table for the Andersen sampler. The airborne fungal concentration (*C*, CFU/m^3^) was calculated using the formula: *C* = *N*/(0.0283 × *T*), where *N* is the corrected colony count (CFU), 0.0283 is the sampling flow rate constant (m^3^/min), and *T* is the sampling time (minutes). The CFU distribution across size stages was used to assess the proportion of respirable and settleable fungi.

### 2.3. Air Sample Collection for Fungal Community Analysis

To obtain fungal community DNA, high-volume air samplers (Model HH02-LS120, Beijing Huaruidean Technology Co., Ltd., Beijing, China) equipped with pre-sterilized quartz fiber filters (Tissuquartz™, 20.32 × 25.4 cm, PALL, Port Washington, NY, USA) were employed. Sampling was conducted at a flow rate of 1000 L/min for 12 continuous hours [23]. Five parallel samplers were deployed simultaneously in each house, and samples from the same growth stage were pooled into one composite sample [8,24]. After sampling, filters were stored at −80 °C until analysis. Prior to DNA extraction, filters were aseptically cut into eight equal segments (weight variation per segment ≤ 1 mg). Fungal material was dislodged from the filter segments via ultrasonic elution in sterile water (ultrasonic power 100 W, duration 15 min, with 30 s intervals) and collected by centrifugation (25,000× *g*, 4 °C, 10 min).

### 2.4. DNA Extraction and ITS High-Throughput Sequencing

Fungal genomic DNA was extracted using the CTAB method [25]. Purity and concentration were measured using a NanoDrop 2000 (Thermo Fisher Scientific, Waltham, MA, USA), with acceptable OD260/280 ratios ranging from 1.8 to 2.0, and integrity was checked on 0.8% agarose gels. Samples failing quality thresholds were re-extracted until standards were met. To analyze fungal community diversity, the ITS1-ITS2 region was amplified using primers ITS1F (5′-CTTGGTCATTTAGAGGAAGTAA-3′) and ITS2R (5′-GCTGCGTTCTTCATCGATGC-3′) [3,26]. The PCR reaction mixture (30 µL total volume) contained 15 µL of 2× PCR Master Mix, 0.2 µM of each primer, and approximately 10 ng of template DNA. Amplification conditions were: initial denaturation at 95 °C for 3 min; followed by 30 cycles of 95 °C for 30 s, 55 °C for 30 s, and 72 °C for 45 s; and a final extension at 72 °C for 10 min. PCR products were visualized via 2% agarose gel electrophoresis and purified using the Qiagen Gel Extraction Kit (28704, Qiagen, Hilden, Germany). Purified amplicons were pooled in equimolar ratios for library construction, and paired-end sequencing (2 × 300 bp) was performed on an Illumina MiSeq PE300 platform (Illumina, San Diego, CA, USA) [18]. Sequencing data were subjected to quality control and assembly by Novogene (Beijing, China). The sequencing data have been deposited in the NCBI GenBank database under BioProject accession number PRJNA1354569.

### 2.5. Data Analysis and Statistical Methods

Raw sequencing reads were processed and quality-filtered in QIIME2 (version 2025.4) to remove low-quality sequences and chimeras. High-quality sequences were clustered into Operational Taxonomic Units (OTUs) at a 97% similarity threshold. Taxonomic annotation was performed using the UNITE database (UNITE General Fungal ITS Database, version 2024_05). Alpha diversity, reflecting community richness and diversity, was assessed using the Chao1 and Shannon indices. Beta diversity, based on Bray–Curtis distances, was analyzed via Principal Component Analysis (PCA) and Non-metric Multidimensional Scaling (NMDS) to reveal community differences across growth stages. The relative abundance of fungal genera was visualized using bar plots and cluster heatmaps. Significance testing was performed using one-way Analysis of Variance (ANOVA) in SPSS (v19.0) and GraphPad Prism (v8.0), with a significance threshold set at *p* < 0.05. All data are presented as mean ± standard deviation (SD).

## 3. Results

### 3.1. Concentration and Size Distribution of Colony Fungal Bioaerosols in Broiler Houses

**Figure 1:**
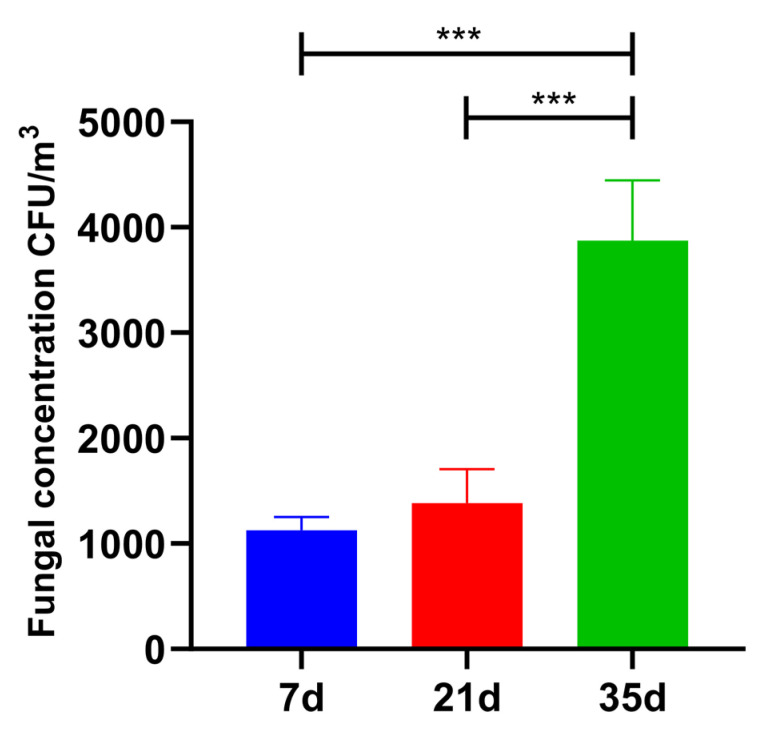
Concentration dynamics of colony fungal bioaerosols in the air of winter confined-cage broiler houses across different broiler ages. *** *p* < 0.001.

The concentration of total colony airborne fungi in the broiler houses increased significantly (*p* < 0.001) throughout the rearing cycle across the three sampling ages (7, 21, and 35 days), as illustrated in Figure 1. The fungal concentration was (1.13 ± 0.13) × 10^3^ CFU/m^3^ at day 7, rose to (1.39 ± 0.32) × 10^3^ CFU/m^3^ at day 21, and reached (3.87 ± 0.56) × 10^3^ CFU/m^3^ at day 35, representing a significant increase compared to both earlier stages.

**Figure 2:**
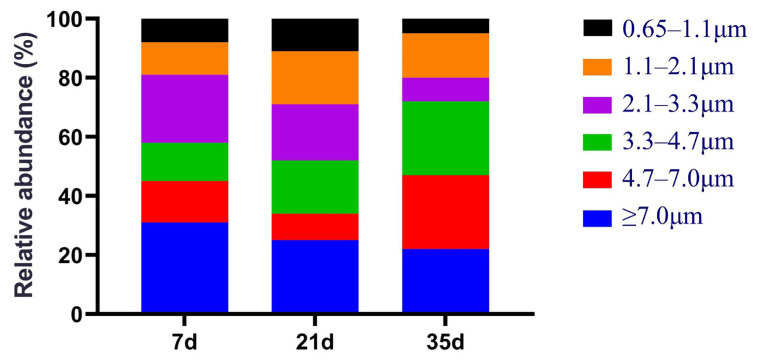
Size distribution characteristics of fungal bioaerosols in the air of winter broiler houses.

The size distribution analysis (Figure 2) revealed that small particles (<4.7 µm) dominated the fungal population. At day 7, these small particles accounted for 55.58% of the total. By day 35, the proportion of large particles (≥4.7 µm) slightly rebounded to 45.65%. Collectively, small-sized fungal particles consistently constituted more than half of the total.

### 3.2. Fungal Community Analysis Based on ITS Gene Sequencing

**Figure 3:**
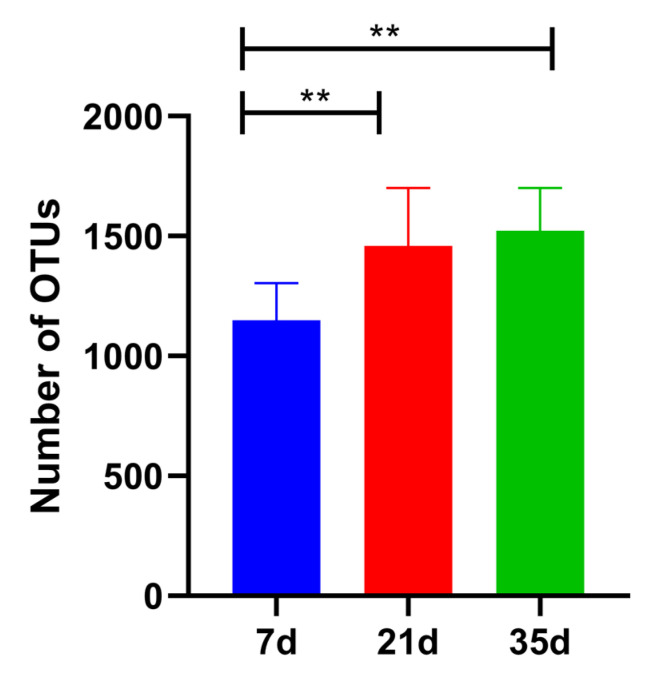
OTU richness in air samples from broiler houses at different ages, as determined by ITS sequencing. ** *p* < 0.01.

A total of 67,432 high-quality sequences were obtained after quality control of the ITS high-throughput sequencing data and were used for fungal community structure analysis. The number of fungal Operational Taxonomic Units (OTUs) exhibited a significant increasing trend (*p* < 0.01) across the different growth stages: 1149 ± 155 at day 7, 1459 ± 241 at day 21, and 1522 ± 179 at day 35 (Figure 3).

### 3.3. Fungal Community Diversity Analysis

**Figure 4:**
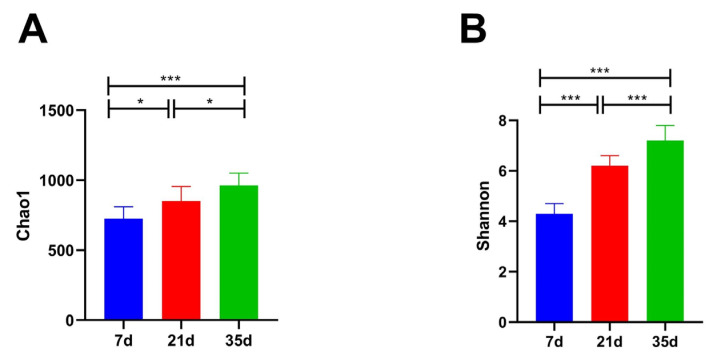
Variations in the alpha diversity indices ((**A**) Chao1 and (**B**) Shannon) of the airborne fungal communities in winter broiler houses across different broiler ages. * *p* < 0.05, *** *p* < 0.001.

As shown in Figure 4, both the Chao1 and Shannon indices of the fungal communities associated with particulate matter in the broiler houses increased significantly (*p* < 0.001) with bird age, indicating a continuous enhancement of community richness and evenness. The Chao1 index increased progressively from 135 ± 18 at day 7, to 249 ± 11 at day 21, and reached 372 ± 23 at day 35. Similarly, the Shannon index rose from 1.3 ± 0.2 at day 7, to 2.8 ± 0.4 at day 21, and further to 3.5 ± 0.1 at day 35.

**Figure 5:**
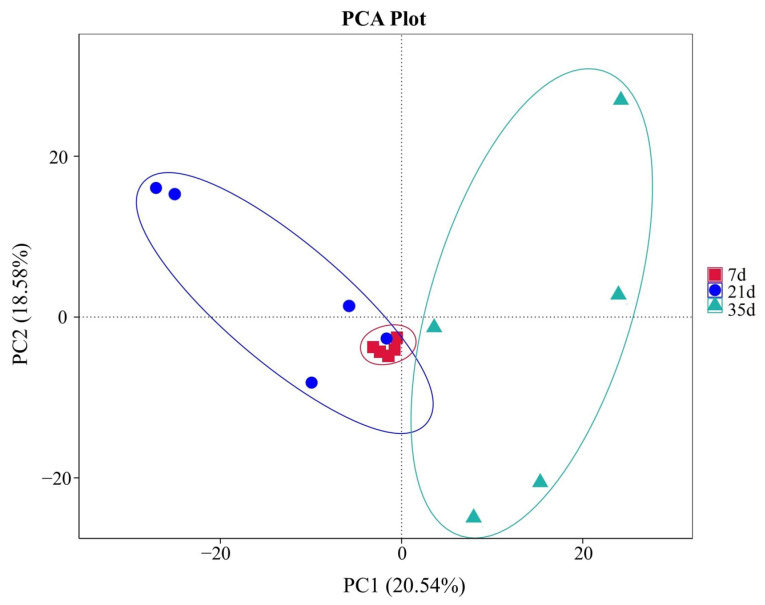
Beta diversity analysis of airborne fungal communities in winter broiler houses, visualized by Principal Component Analysis (PCA) based on Bray–Curtis distances.

Principal Component Analysis (PCA) revealed a clear separation (*p* < 0.05) among the samples from the three different ages (7, 21, and 35 days), with data points forming distinct clusters for each stage (Figure 5).

### 3.4. Fungal Community Composition and Succession Characteristics

**Figure 6:**
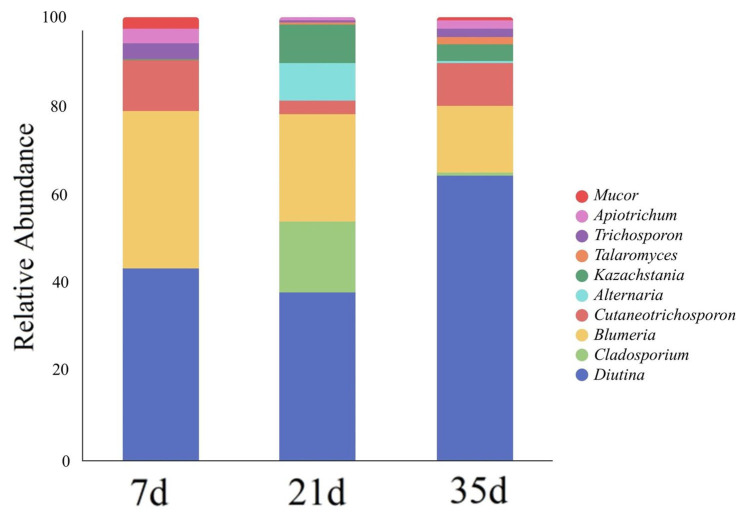
Relative abundance composition of airborne fungal communities at the genus level in broiler houses across different ages.

**Figure 7:**
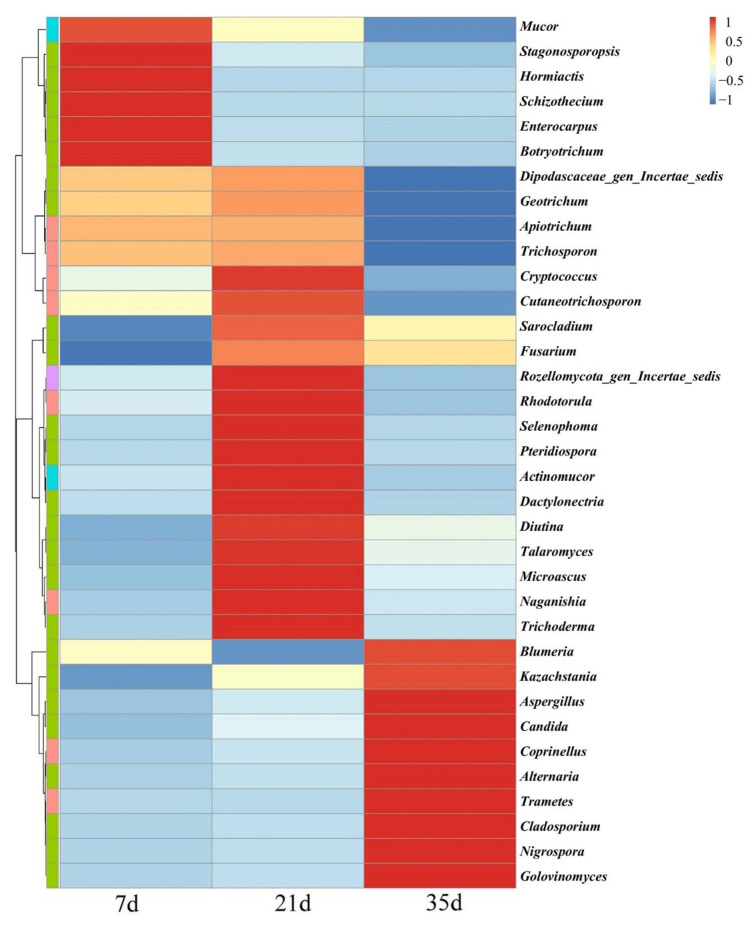
Cluster heatmap of airborne fungal communities in broiler houses across different ages.

As shown in Figure 6 and Figure 7, the fungal community at day 7 was dominated by yeast-type fungi, mainly *Diutina* (42.58%), *Blumeria* (36.45%), and *Cutaneotrichosporon* (12.76%), with additional contributions from *Trichosporon* (4.06%), *Apiotrichum* (4.48%), and *Mucor* (3.85%).

A shift in community structure was observed at day 21, marked by notable changes in the relative abundance of several genera. The abundance of *Diutina* decreased to 38.35%, while *Cladosporium* (19.87%), *Alternaria* (14.85%), and *Kazachstania* (15.31%) became increasingly prevalent.

By day 35, the dominant genera included *Diutina* (63.34%), *Blumeria* (16.88%), *Cutaneotrichosporon* (11.47%), *Kazachstania* (5.56%), *Apiotrichum* (2.48%), *Trichosporon* (2.36%), *Talaromyces* (2.18%), *Mucor* (1.53%), *Cladosporium* (1.47%), and *Alternaria* (1.07%). The persistent presence and relatively balanced proportions of multiple filamentous fungal genera indicated a significantly enhanced level of community diversity and more complex ecological functionality.

Overall, the community structure demonstrated a transition from a dominance of specific yeast-like fungi in the early stage to a community increasingly influenced by filamentous fungi in the later stage.

Notably, several zoonotic pathogenic fungal genera were detected in the broiler house bioaerosols, including *Geotrichum*, *Cryptococcus*, *Fusarium*, *Trichoderma*, *Aspergillus*, *Candida*, *Alternaria*, and *Cladosporium*, all listed in the “Catalog of Pathogenic Microorganisms Transmissible from Animals to Humans” (2023).

## 4. Discussion

This study provides a comprehensive characterization of airborne fungal bioaerosols in winter-confined commercial broiler houses by integrating concentration dynamics, aerodynamic particle-size distribution, and ITS-based community succession across three key rearing stages (7, 21, and 35 days). Unlike long-cycle poultry production systems, commercial broiler production is defined by a short growth period, high stocking density, rapid biomass turnover, and intensive ventilation control. Under such conditions, airborne fungal dynamics are expected to respond rapidly to changes in animal metabolism, manure accumulation, and environmental disturbance. Our results reveal a clear stage-dependent pattern of fungal aerosol amplification that is tightly coupled with the broiler production cycle, providing system-specific insights into airborne microbial risks in winter broiler houses.

The progressive increase in culturable airborne fungal concentration from day 7 to day 35 indicates that fungal accumulation in broiler houses is not a linear background process but rather a rapid amplification phenomenon driven by short-cycle intensive rearing. As broilers age, metabolic heat production, feed intake, and manure output increase sharply, resulting in accelerated release of organic substrates and particulate carriers into the air. Under winter-confined conditions, ventilation rates are deliberately reduced to maintain thermal stability, which limits dilution and removal of airborne particles. The combination of increased emission intensity and restricted air exchange creates favorable conditions for fungal persistence and accumulation [1,16,27,28]. Importantly, the pronounced increase observed at the finishing stage (35 days) suggests that the late broiler phase represents a critical window of elevated airborne fungal burden, which is particularly relevant for production performance and biosecurity management.

In addition to concentration changes, particle-size distribution analysis revealed that small-sized fungal aerosols (<4.7 μm) consistently dominated across all stages, accounting for more than half of the total fungal load. This finding has important implications for both transmission behavior and health risk. In intensive broiler houses, fine particles are continuously generated through feed handling, bird movement, feather shedding, and manure disturbance. These particles readily act as carriers for fungal spores, facilitating their suspension and long-range transport [3,8]. The predominance of respirable-sized fungi during the early and middle stages indicates that fungal aerosols in broiler houses are not only abundant but also aerodynamically optimized for deep respiratory penetration [1,29]. Although a slight increase in larger particles was observed at day 35, likely due to particle agglomeration associated with increased organic dust and feather debris, respirable fractions remained dominant, underscoring the persistent inhalation risk throughout the production cycle.

The health relevance of this size profile is further reinforced by the taxonomic composition of the fungal community. Several dominant genera detected in this study, including *Aspergillus*, *Cladosporium*, *Alternaria*, *Fusarium*, *Candida*, and *Cryptococcus*, are well known for producing spores or fragments within respirable size ranges [10,20,30,31]. The increasing proportion of small-sized fungi during the early and middle stages (7 and 21 days) reflects a tendency for fungi to produce highly transmissible, respirable spores during this phase. The slight rebound in larger particles at day 35 might be attributed to spore agglomeration caused by increased feed residue accumulation and feather dander in the later rearing phase. The health relevance of airborne fungal bioaerosols is closely linked to both fungal species composition and spore aerodynamic characteristics. In the present study, several dominant genera identified in winter poultry houses, including *Aspergillus*, *Cladosporium*, *Alternaria*, *Fusarium*, *Candida*, and *Cryptococcus*, are well recognized for producing spores or propagules within respirable size ranges. Previous studies have shown that spores of *Aspergillus* and *Cladosporium* typically range from 2 to 5 µm, while *Alternaria* and *Fusarium* can also generate fragmented particles capable of remaining airborne and penetrating the lower respiratory tract. Consistent with these reports, our particle size distribution analysis demonstrated a predominance of small-sized fungal aerosols (<4.7 µm) across all production stages, particularly during the early and middle periods. Such fine particles can remain suspended for prolonged durations and deposit in the bronchiolar and alveolar regions, thereby increasing the potential for respiratory irritation, allergic sensitization, and inflammatory responses. Under winter confined conditions with limited ventilation, the sustained presence of respirable fungal aerosols may therefore contribute to elevated occupational exposure risks for farm workers and respiratory health challenges in poultry [20,32]. Consequently, the dominance of small-sized fungi observed in this study essentially signals an “enhanced airborne adaptability,” posing a threat to both poultry health and constituting a persistent occupational exposure risk for farm workers.

ITS-based sequencing further revealed pronounced stage-dependent succession of airborne fungal communities. At day 7, the community was dominated by yeast-like fungi such as *Diutina* and *Blumeria*, which are typically associated with readily available nutrients and minimal environmental complexity. During this early stage, manure accumulation is limited and organic substrates are relatively simple, favoring fast-growing yeasts capable of rapid colonization. By day 21, increased manure deposition, feed residues, and fluctuating microclimatic conditions promoted the enrichment of filamentous fungi such as *Cladosporium* and *Alternaria*. By day 35, community diversity increased markedly, with mold genera characterized by strong sporulation capacity and environmental tolerance becoming more prominent. This rapid succession reflects the accelerated ecological turnover inherent to short-cycle broiler production, where environmental conditions evolve over weeks rather than months.

This successional trajectory aligns well with the functional successional replacement model in microbial ecology [13,30,33]. In the early stages of system establishment, yeast-like fungi, which are closely associated with host activity, exhibit rapid growth rates, and are sensitive to environmental perturbations, hold a natural advantage, hence their high abundance at day 7. As substrate structure, microclimate, and aerodynamic disturbances gradually change, filamentous molds—characterized by high spore yield, desiccation tolerance, and ability to remain airborne for extended periods—expand rapidly and dominate the aerial niche. Multiple studies confirm a highly consistent direction for airborne fungal community succession in poultry houses: a shift from “vegetative growth-oriented” to “dispersal-oriented” communities as manure accumulates, airflow disturbance intensifies, humidity fluctuations increase, and dust levels rise [7,30].

Various zoonotic pathogenic fungi were detected in air samples across all three stages, including *Aspergillus*, *Fusarium*, *Cladosporium*, *Alternaria*, *Candida*, and *Cryptococcus*. These genera are listed as significant health concerns by the World Health Organization and China’s “Catalog of Pathogenic Microorganisms Transmissible from Animals to Humans,” carrying defined health risks: they can cause respiratory diseases and immunosuppression in poultry, affecting production performance, and can also lead to chronic rhinitis, asthma, and allergic alveolitis in farm workers via airborne transmission [34,35]. Some, like *Aspergillus*, can cause opportunistic infections [10,36,37,38]. Notably, *Fusarium* species can produce various mycotoxins, and exposure to airborne mycotoxins is an emerging exposure pathway of international concern [8]. Furthermore, studies have demonstrated that fungal spores can adsorb antibiotic resistance genes and participate in resistance dissemination networks, positioning airborne fungi as potential nodes in antimicrobial resistance spread [39]. This evidence indicates that airborne fungal pollution in broiler houses is no longer solely an animal health issue but a cross-boundary public health challenge involving flocks, farm workers, and the environment, bearing typical One Health characteristics.

In addition to the taxonomic succession observed at the genus level, the richness and diversity metrics further supported a stage-dependent restructuring of the airborne fungal community. The significant increases in OTU richness from day 7 to day 35 (Figure 3), together with the rising Chao1 and Shannon indices (Figure 4), suggest a progressive diversification of fungal sources and ecological niches in the house air as the production cycle progressed. Moreover, the clear separation of samples by growth stage in the PCA (Figure 5) indicates that broiler age and the associated micro-environmental shifts acted as strong determinants of community structure. This pattern is plausibly driven by the combined effects of increasing manure- and feed-derived substrates, enhanced dust and feather dander loading, and winter ventilation restriction, which collectively reshape aerosolization intensity and the temperature–humidity regime, thereby promoting community turnover and enrichment across the rearing stages. The fungal community changes revealed in this study result from the interplay of multi-layered ecological regulatory mechanisms within the poultry house aerial system, including ventilation dynamics, microclimatic stability, particulate matter load, and animal activity. Winter temperature control strategies in broiler houses typically maintain a range of 21–33 °C, which falls within the optimal growth temperature range for many fungi, inadvertently turning the house into an optimal fungal chamber during winter [37,40]. Simultaneously, humidity exhibits a typical “phasic fluctuation” pattern in winter—increased humidity promotes mold growth, while decreased humidity facilitates spore drying, release, and dispersal. This “dual-driver mode” accelerates the ecological transition of fungi from the growth phase to the dispersal phase [41]. Additionally, air disturbances caused by bird activity and feeding behavior further propel spores into suspension, enabling continuous ecological redistribution of airborne fungi. The significant community differences among the 7, 21, and 35-day stages, as shown by our PCA, directly reflect this air dynamic-driven community differentiation and show significant coupling with broiler behavioral rhythms.

In summary, this study demonstrates that airborne fungal bioaerosols in winter broiler houses are governed by a rapid, stage-dependent ecological amplification process shaped by intensive short-cycle production. The coupled effects of concentration accumulation, respirable particle dominance, and accelerated community succession distinguish broiler systems from long-cycle poultry operations and underscore the need for tailored control strategies. Targeted interventions should prioritize the finishing stage, when fungal burden peaks, and focus on optimizing winter ventilation, reducing organic dust sources, and implementing routine monitoring of respirable fungal fractions. From a One Health perspective, such measures are essential for protecting flock health, safeguarding occupational safety, and mitigating environmental dissemination of airborne fungal hazards.

It should be noted that the present study is conceptually related to our previously published work [42]; however, the two studies differ substantially in terms of poultry model, production system, and scientific focus. That study investigated fungal aerosol exposure in Taihang chickens, an indigenous breed characterized by a long production cycle, with primary emphasis on occupational exposure and One Health–related risk assessment. In contrast, the current study focuses on commercial broiler chickens raised under a short, intensive production cycle, which is fundamentally different in stocking density, growth rhythm, manure accumulation rate, and winter ventilation management. These system-level differences result in distinct airborne fungal microecological dynamics. Accordingly, the present work emphasizes stage-dependent fungal accumulation, particle size refinement, and community succession under intensive broiler production conditions, providing system-specific insights that were not addressed in the previous study. Several limitations of the present study should be acknowledged. First, sampling was conducted during a single winter production cycle, which may not fully capture seasonal variability in airborne fungal concentrations and community composition. Second, although colony analysis combined with ITS-based high-throughput sequencing provided complementary insights into fungal bioaerosols, non-colony fungi and fungal metabolites were not directly assessed. Third, this study focused on environmental characterization and did not include direct exposure assessment or health outcome measurements in poultry or farm workers. Future studies integrating multi-season sampling, functional profiling, and exposure–response analysis will be essential to further elucidate the health risks associated with airborne fungal bioaerosols in confined poultry production systems.

## 5. Conclusions

This study systematically investigated the concentration dynamics, particle size distribution, and ITS-based community succession of airborne fungal bioaerosols in winter confined-cage broiler houses across three key growth stages (7, 21, and 35 days). Our findings reveal a stage-specific ecological amplification of fungal aerosols within this typical high-density poultry environment. The results demonstrated a significant accumulation of fungal bioaerosols as the rearing cycle progressed, with concentrations at day 35 approaching the high-exposure risk thresholds reported internationally. The consistent dominance of small-sized fungi (<4.7 μm) underscores their potential for deep respiratory deposition and long-distance airborne transmission, highlighting a dual health risk to both the poultry and farm personnel.

ITS sequencing further delineated a clear community succession from early-stage yeast dominance to late-stage assemblages dominated by filamentous fungi. The detection of several crucial zoonotic pathogenic genera (e.g., *Aspergillus*, *Cladosporium*, *Candida*) reflects a characteristic One Health exposure risk profile under the coupled conditions of restricted ventilation and high dust load in winter poultry houses. This study establishes an aerial transmission ecological model of “concentration accumulation—particle size refinement—community restructuring” for fungal bioaerosols in winter confined-cage systems, emphasizing their role as critical ecological drivers and potential airborne bio-safety hazards in poultry production environments.

Our results provide not only essential data for understanding the ecology of fungal aerosols in poultry houses but also a scientific basis for precisely formulating winter ventilation strategies, improving manure management, developing airborne microbial monitoring technologies, and establishing comprehensive aerial biosafety protocols for poultry farms. Future research should prioritize elucidating the mechanisms of combined exposure involving fungi, bacteria, viruses, particulate matter, and mycotoxins. A concerted effort integrating environmental engineering, animal health, and public health perspectives is needed to develop targeted intervention strategies. This integrated approach is fundamental to building a unified “flock–personnel–environment” health protection framework and for promoting a poultry production industry characterized by high health standards, minimized risks, and sustainable development.

## Data Availability

All original contributions of this study are contained within the article, further details are available from the corresponding authors upon request.
